# Supplementary material for: OBERON3 and SUPPRESSOR OF MAX2 1-LIKE proteins form a regulatory module driving phloem development
Source: Nat Commun. 2023 Apr 14;14:2128. doi: 10.1038/s41467-023-37790-5 (PMC10104830; doi:10.1038/s41467-023-37790-5)
Supplement: Supplementary file 1 — Supplementary information [file 41467_2023_37790_MOESM1_ESM.pdf]

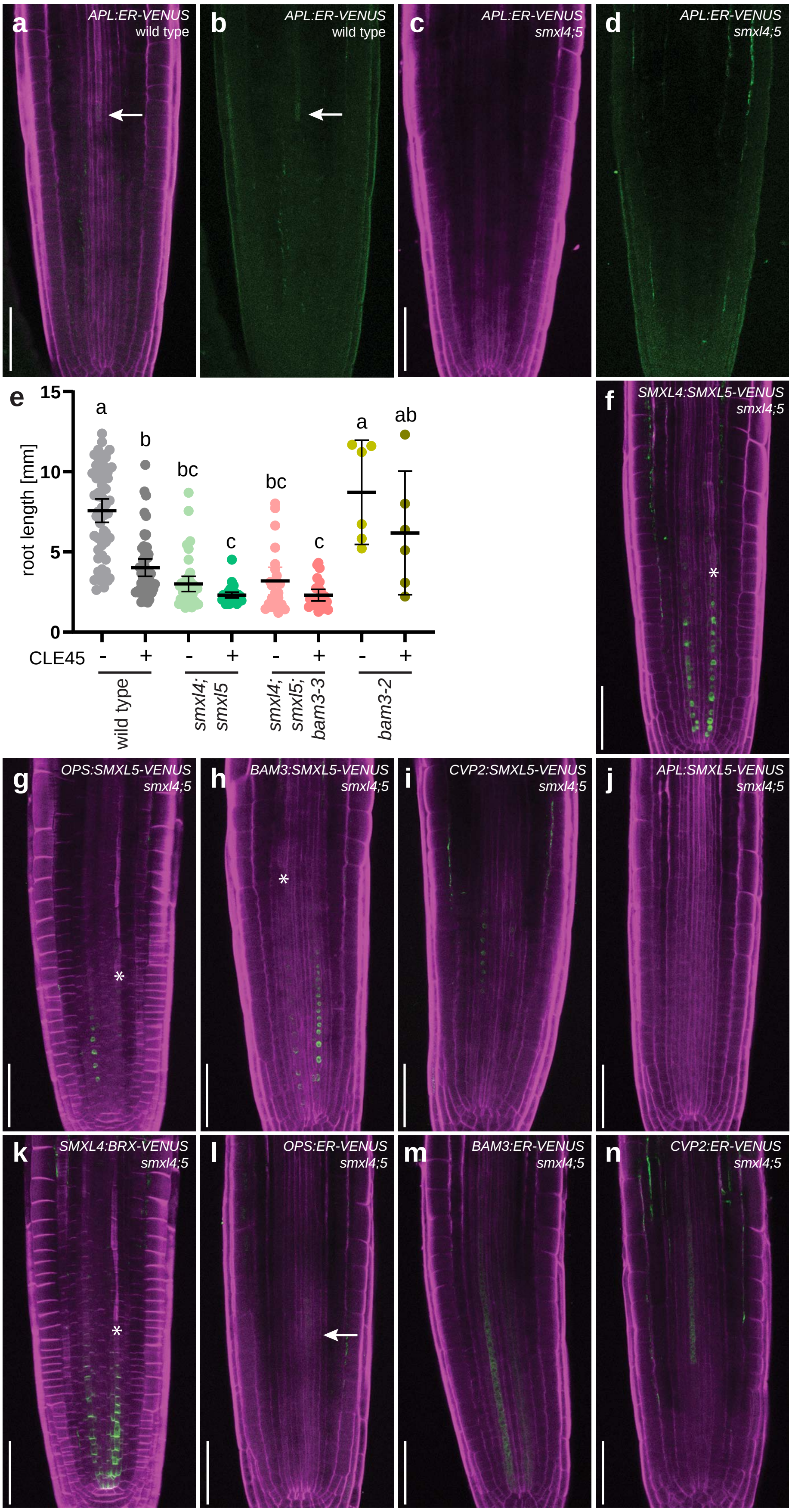

Supplementary Fig. 1: Expression of SMXL5-VENUS protein fusions in smxl4;smxl5 mutants in comparison to promoter reporters.

a – d Detection of *APL:ER-VENUS* reporter activity in wild type (j, k) and *smxl4;smxl5* mutants (l, m). k and m show the same root tip as in j and l, respectively, without the counter stain signal. Scale bars in all pictures represent 50 µm. Arrows in j and k point to a weakly detectable reporter-derived signal.

e Root length measurements in different genetic backgrounds in the presence and absence of exogenous CLE45 five days after germination. Results from one representative experiment out of two independent experiments are shown (see Source Data File for exact sample numbers). Mean values were analyzed by one-way ANOVA with post-hoc Tukey HSD (95 % CI). Statistical groups are indicated by letters.

f – j Expression of SMXL5-VENUS protein fusions under the control of different heterologous promoters. Differentiated SEs are indicated by asterisks.

k Detection of the BRX-VENUS protein fusion expressed under the control of the *SMXL4* promoter. Differentiated SEs are indicated by the asterisk.

l – n Detection of *OPS:ER-VENUS*, *BAM3:ER-VENUS*, and *CVP2:ER-VENUS* reporter activities in *smxl4;smxl5* mutants. Arrow in l points to a weakly detectable reporter-derived signal.

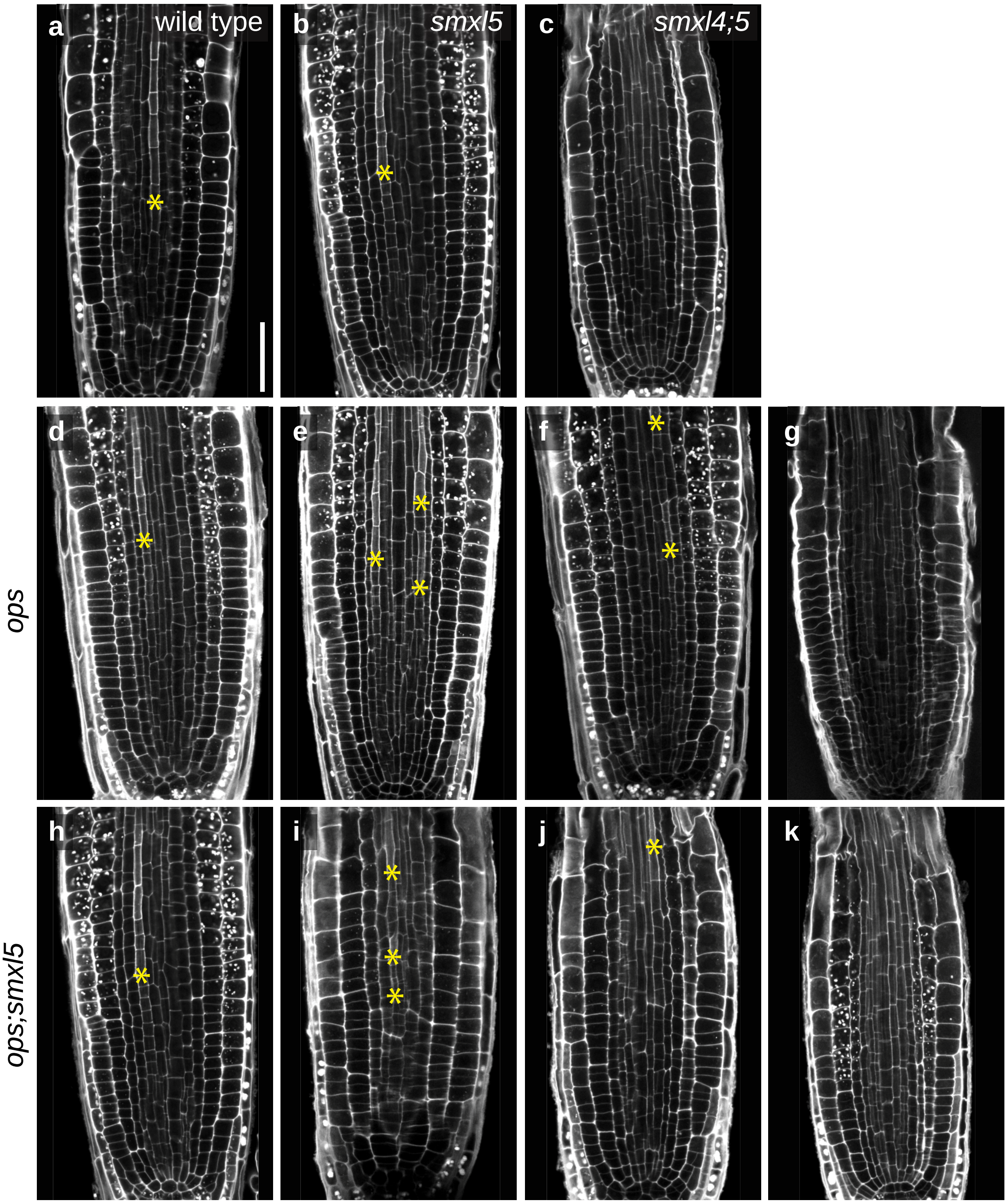

Supplementary Fig. 2: Characterization of phloem development in *smxl* and *ops* mutants.
a – k Phloem development is monitored in wild type (a), *smxl5* (b), *smxl4;smxl5* (c), *ops* (d-g) and *ops;smxl5* (h-k) mutant backgrounds by confocal analysis of mPS-PI-stained root tips. Asterisks indicate the most apical appearance of differentiated SEs. In case 'gaps' are observed, more than one asterisk is depicted. At least 18 samples were analysed for each group with similar results.

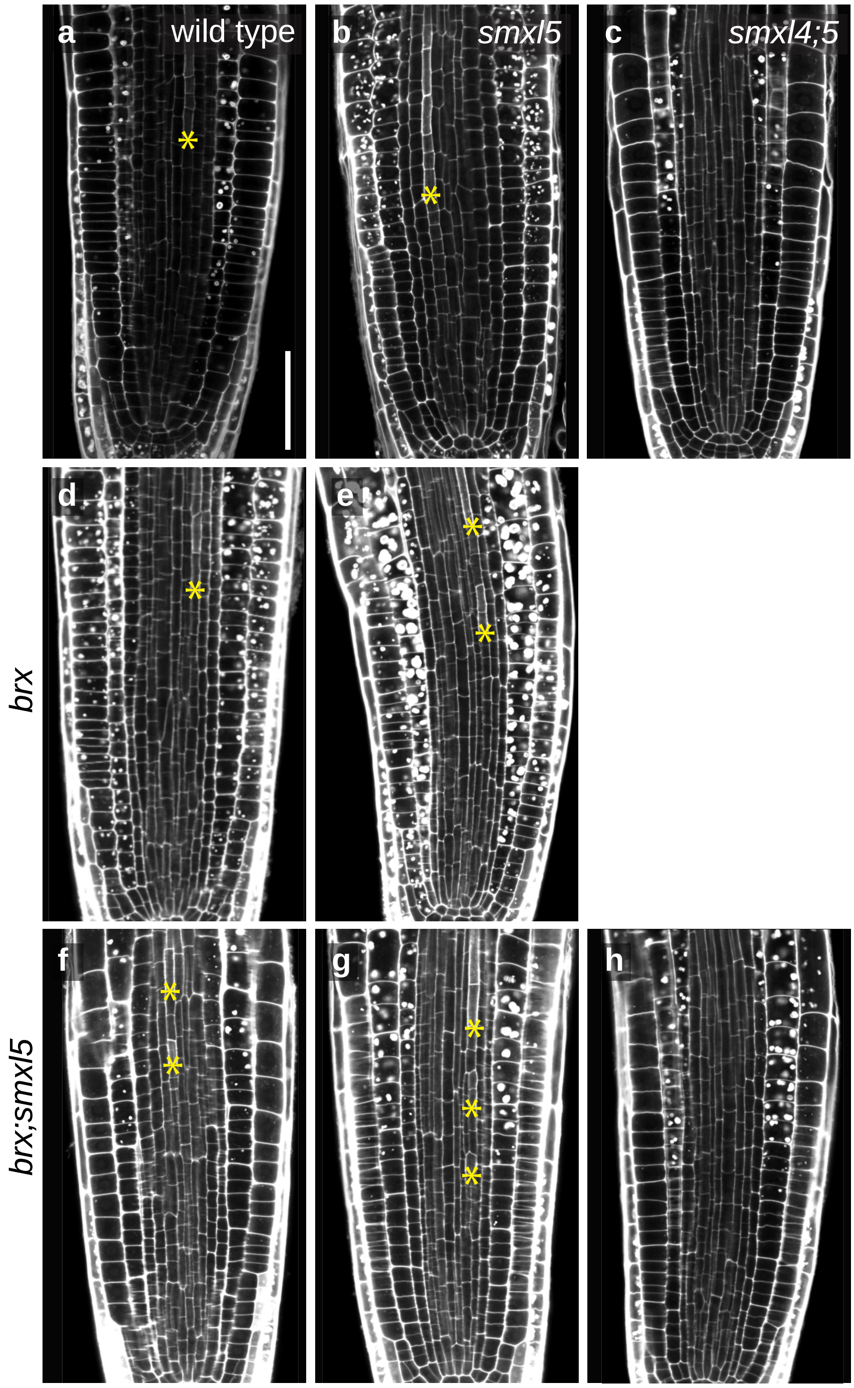

Supplementary Fig. 3: Characterization of phloem development in *smxl* and *brx* mutants.
a – h Phloem development is monitored in wild type (a), *smxl5* (b), *smxl4;smxl5* (c), *brx* (d-e) and *brx;smxl5* (f-h) mutant backgrounds by confocal analysis of mPS-PI-stained root tips. Asterisks indicate the most apical appearance of differentiated SEs. In case 'gaps' are observed, more than one asterisk is depicted. At least 18 samples were analysed for each group with similar results.

a

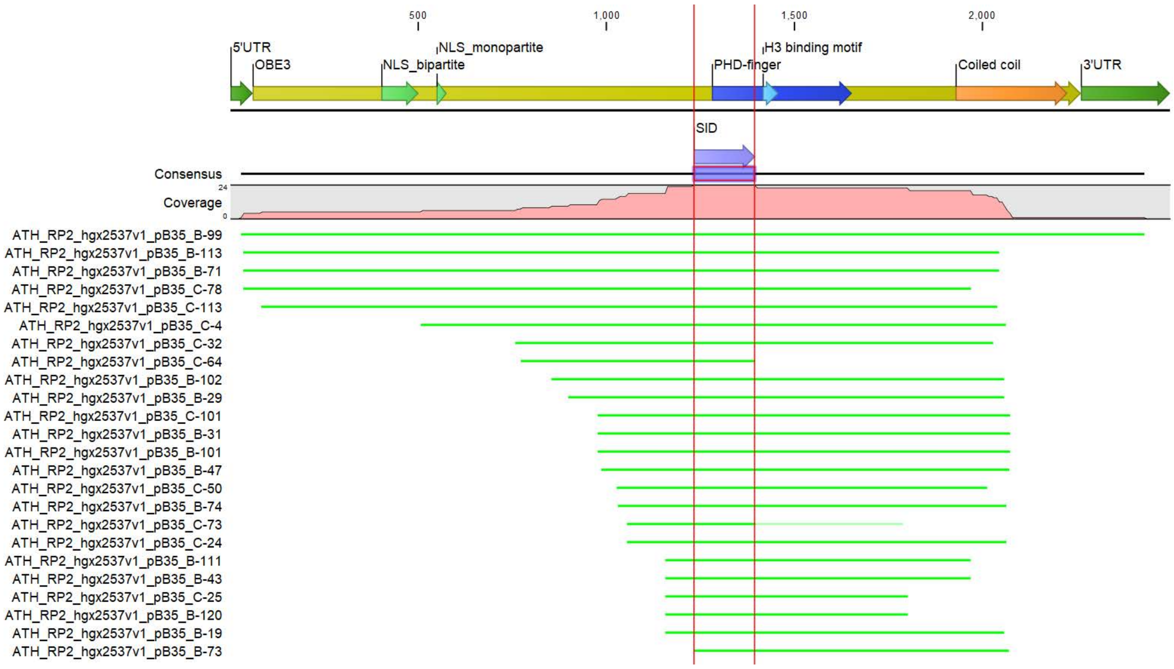

b

-IRIPMNELVEIFLFLRCRNVNCKSLLPVDDCECKICSNNKGFCSSCMCPVCLRF-

Supplementary Fig. 4: *OBE3* cDNA clones isolated when screeing for SMXL5 interactors using the Yeast-Two-Hybrid system.

a Alignment of isolated clones (in green) to the full *OBE3* cDNA sequence (top) using CLC Main Workbench Version 7.6.1 (CLC Bio Qiagen, Aarhus, Denmark). Protein domains predicted for OBE3 are indicated above. The sequence area present in all isolated clones (selected interacting domain, SID) is marked by a purple arrow and flanked by red lines. The OBE3 protein contains two predicted nuclear localization signals (NLS) identified by the cNLS Mapper[1] (NLS_bipartite and NLS_monopartite, light green arrows), a PHD-finger domain (blue arrow) with a histone 3 (H3) binding motif (light blue arrow) and a coiled coil domain (orange arrow) predicted by InterPro (EMBL-EBI, Cambridgeshire, UK). The *OBE3* open reading frame is flanked by a 5’ and a 3’ untranslated region (5’UTR and 3’UTR, green arrows), respectively.

b Amino acid sequence encoded by the SID sequence, present in all isolated clones.

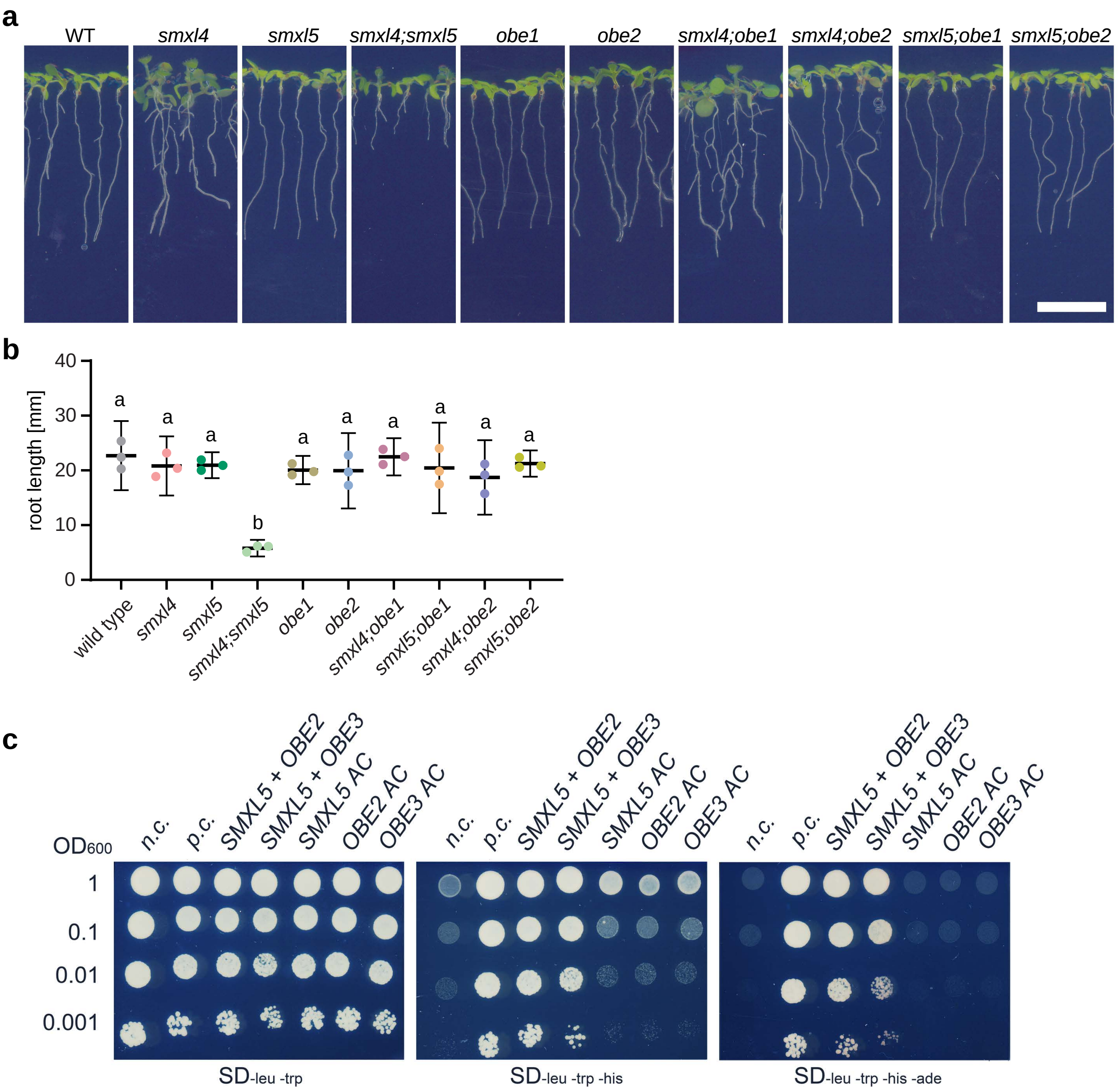

Supplementary Fig. 5: *SMXL4* or *SMXL5* genes do not genetically interact with *OBE1* or *OBE2* genes and protein interaction anaylsis using the yeast-two-hybrid system.

a 10 day-old wild type, *smxl4*, *smxl5*, *smxl4;smxl5*, *obe1*, *obe2*, *smxl4;obe1*, *smxl4;obe2*, *smxl5;obe1*, *smxl5;obe2* seedlings are shown from left to right. Scale bar represents 1 cm.

b Quantification of root lengths depicted in a. Mean values of three independent experiments (see Source Data File for exact sample numbers) were analyzed by a one-way ANOVA with post-hoc Tukey HSD (95 % CI). Statistical groups are indicated by letters.

c Protein interaction anaylsis using the yeast-two-hybrid system. The SMXL5 protein was expressed in yeast fused to the GAL4 DNA binding domain and OBE proteins fused to the GAL4 activation domain. All strains contained AD- and BD-expressing plasmids either alone or fused with SMXL5, OBE3 or control proteins, respectively. AC: Self-activation control. Growth on SD-leu-trp indicate the presence of both plasmids, growth on SD-leu-trp-his and SD-leu-trp-his-ade medium indicate the presence of plasmids and protein interaction. OD600 values indicate the density of spotted yeast cultures. Positive control (p. c.): yeast atrain expressing SV40 large-T-antigen fused to GAL4-AD and p53 fused to GAL4-BD, negative control (n. c.): yeast strain expressing lamin C fused to GAL4-BD and the GAL4-AD.

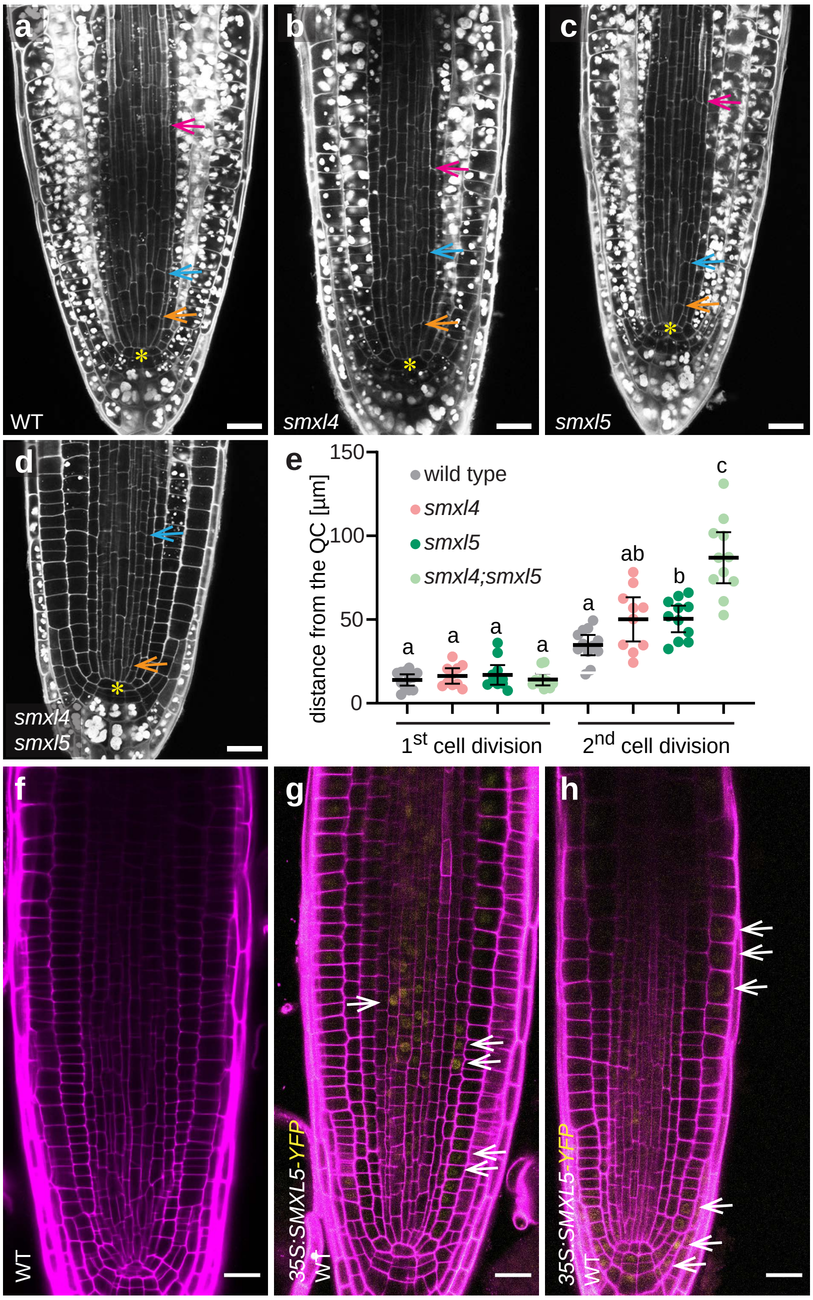

Supplementary Fig. 6: Tissue composition is neither altered in *smxl4* and *smxl5* single mutants nor in *35S:SMXL5-YFP* plants

a – d 2 day-old mPS-PI-stained root tips of wild type (a), *smxl4* (b), *smxl5* (c) and *smxl4;smxl5* (d) plants. Differentiating SEs were observed in wild type, *smxl4* and *smxl5* (pink arrows). Tangential cell divisions are marked by orange and blue arrows. The QC is indicated by a yellow asterisk. Scale bars represent 20 µm. At least 10 samples were analysed for each group with similar results.

e The distance of the first and second tangential division from the QC for plants shown in a-d was quantified (see Source Data File for exact sample numbers). Statistical groups marked by letters were determined by one-way ANOVA with post-hoc Tamhane-T2 (95 % CI). Distances of 1st cell divisions and 2nd cell divisions were compared independently.

f – h Comparision of RAM anatomy of wild type (wt) and *35S:SMXL5-YFP* plants. Although the YFP signal was weak, nuclear localization of the SMXL5-YFP protein could be observed in various cell types (arrows). No obvious difference between RAM anatomies were detected. Two RAM examples of *35S:SMXL5-YFP* plants are shown.

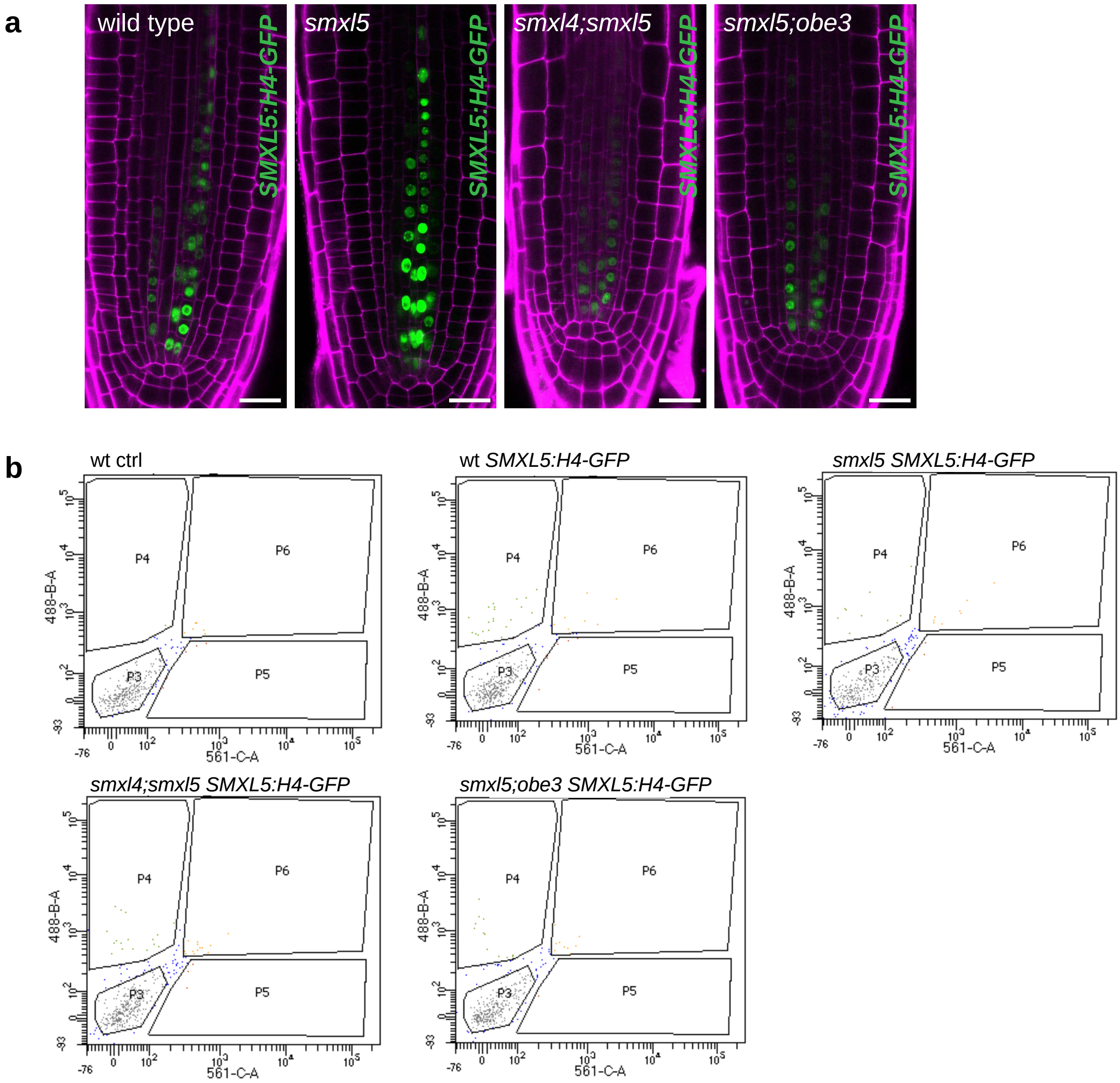

Supplementary Fig. 7: Activity of the *SMXL5:H4-GFP* transgene in wild type, *smxl5*, *smxl4;smxl5* and *smxl5;obe3* backgrounds and sorting of GFP-positive and GFP-negative nucleus fractions

a Activity of the *SMXL5:H4-GFP* transgene in root tips of wild type, *smxl5*, *smxl4;smxl5* and *smxl5;obe3* seedlings two days after germination. Scale bars represent 20 µm. At least 10 sampes were analysed for each group with similar results.

b Sorting plots displaying gates for identifying GFP-positive (P4) and GFP-negative (P3) nuclei comparing wild type without transgene (wt ctrl) and wild type, *smxl5*, *smxl4;smxl5* and *smxl5;obe3* plants carrying the *SMXL5:H4-GFP* transgene. 10,000 sorting events were processed for display in all cases. P3 fractions included 2.2 to 2.9 % of all events and P4 fractions 0.1 to 0.2 % (0 % for wild type without the transgene). X-axes indicate RFP fluorescence (561 nm), Y-axes indicate GFP fluorescence (488 nm).

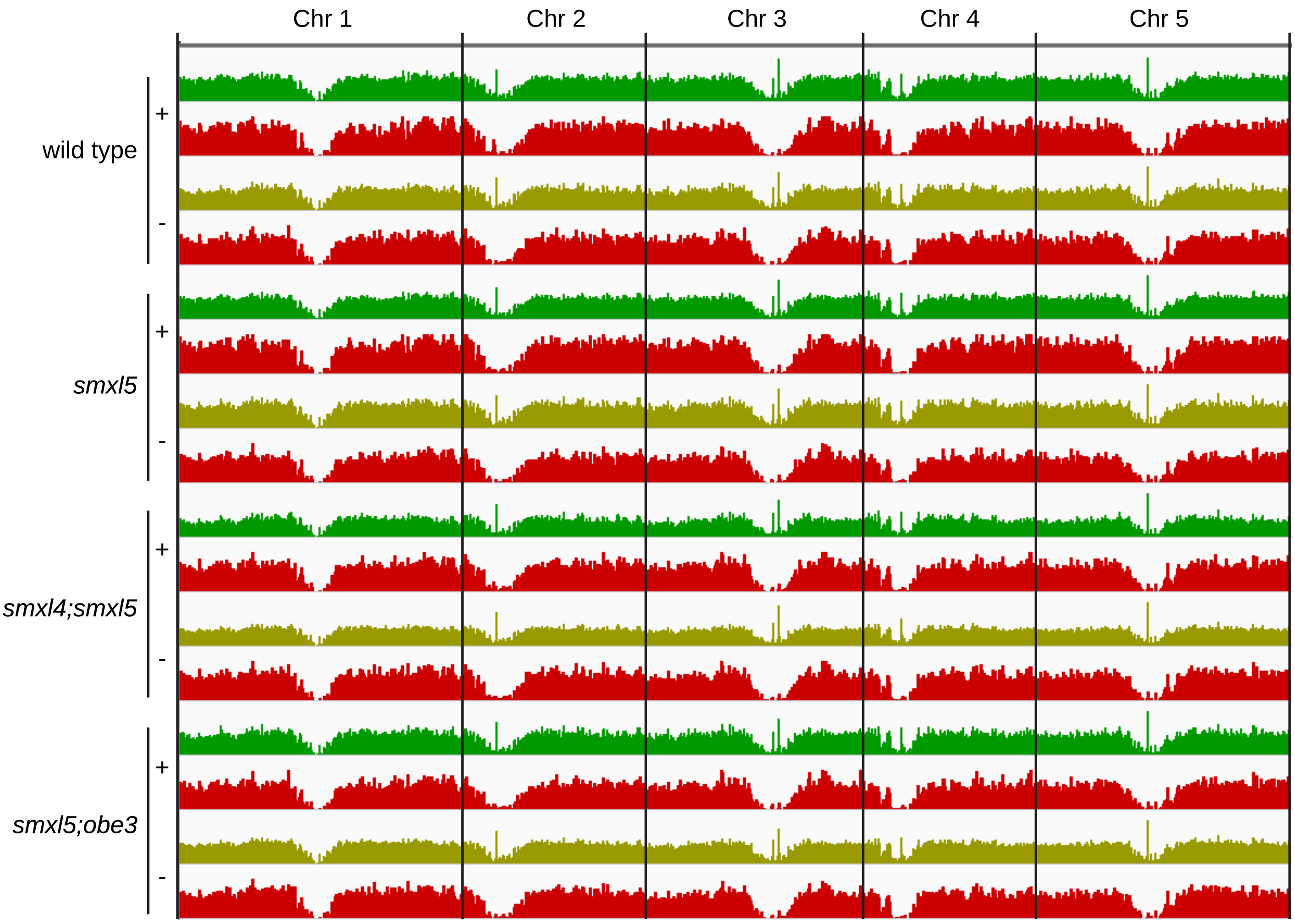

Supplementary Fig. 8: ATAC-seq analysis comparing GFP-positive and GFP-negative samples from wild type, *smxl5*, *smxl4;smxl5* and *smxl5;obe3* seedlings

Profile of read alignment (dark and light green) and OCR detection (red) across all chromosomes for all samples processed. Dark green: GFP-positive samples. Light green: GFP-negative samples. Centromeric regions are indicated by reduced read alignment and OCR detection.

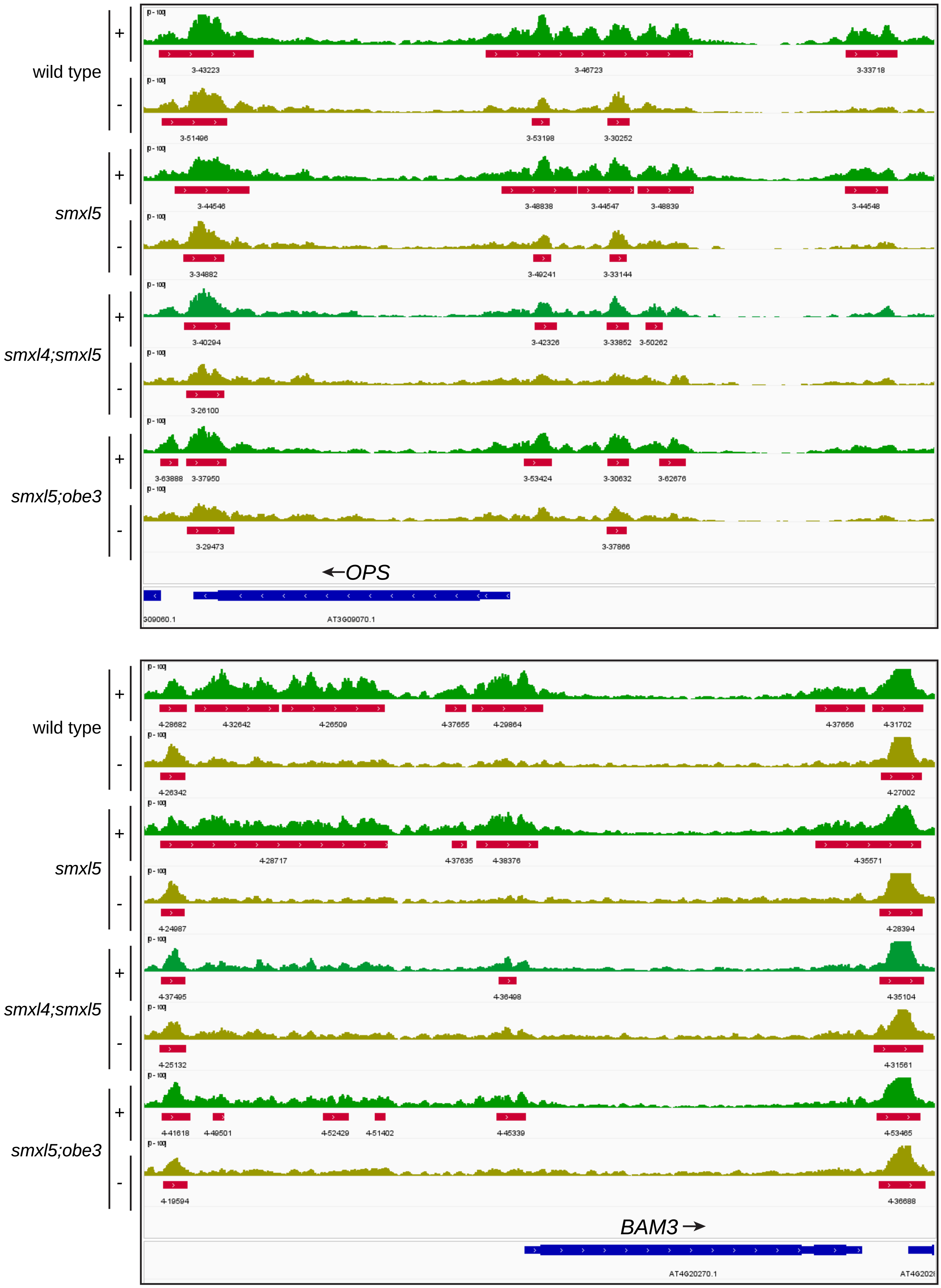

Supplementary Fig. 9: Chromatin conformation profile for *OPS* and *BAM3* gene regions.
Profile of read alignment (dark and light green) and OCR detection (red) for all samples are shown. Dark green: GFP-positive samples. Light green: GFP-negative samples. Gene structures are indicated at the bottom (dark blue).

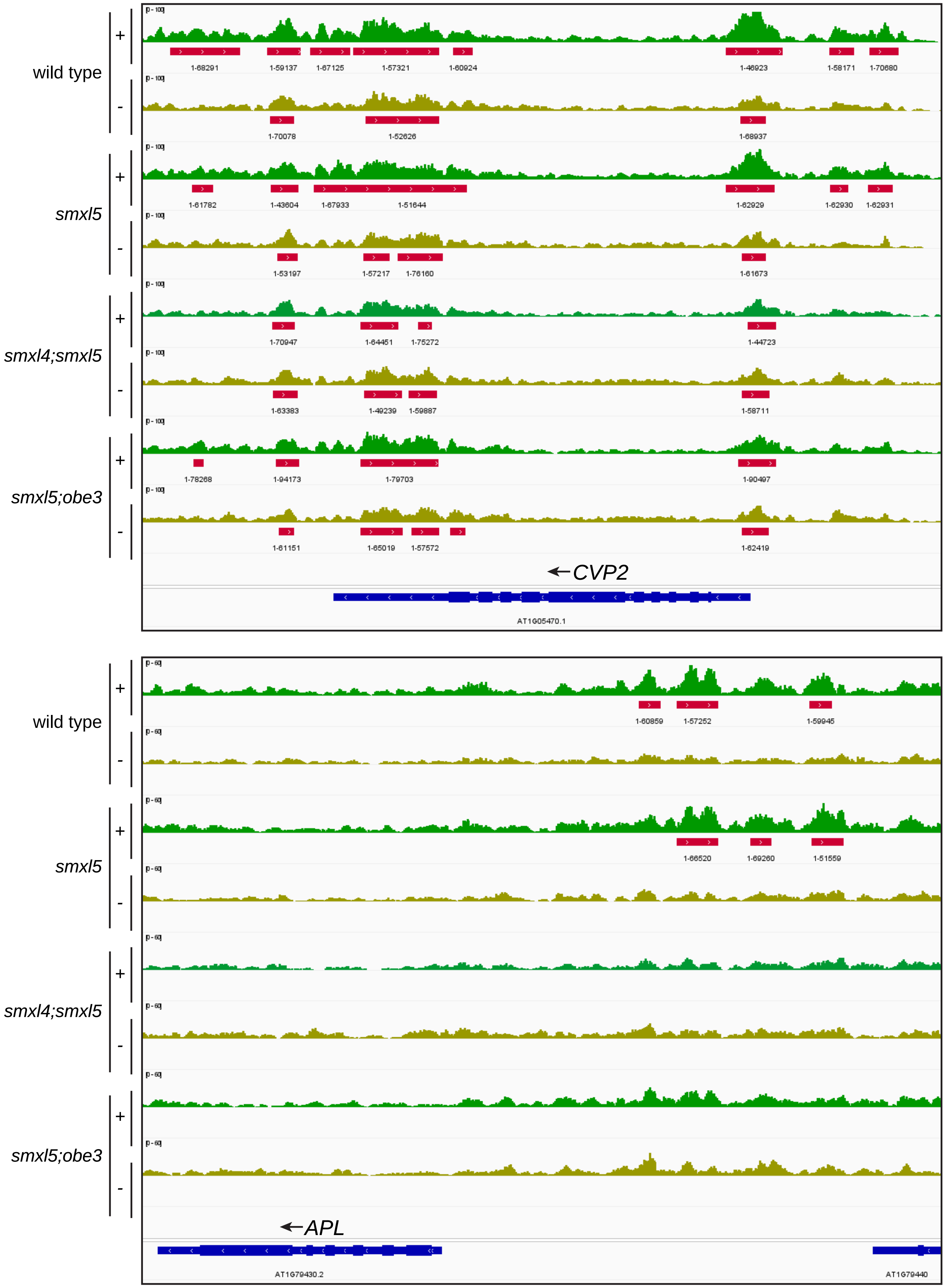

Supplementary Fig. 10: Chromatin conformation profile for *CVP2* and *APL* gene regions.
Profile of read alignment (dark and light green) and OCR detection (red) for all samples are shown. Dark green: GFP-positive samples. Light green: GFP-negative samples. Gene structures are indicated at the bottom (dark blue).

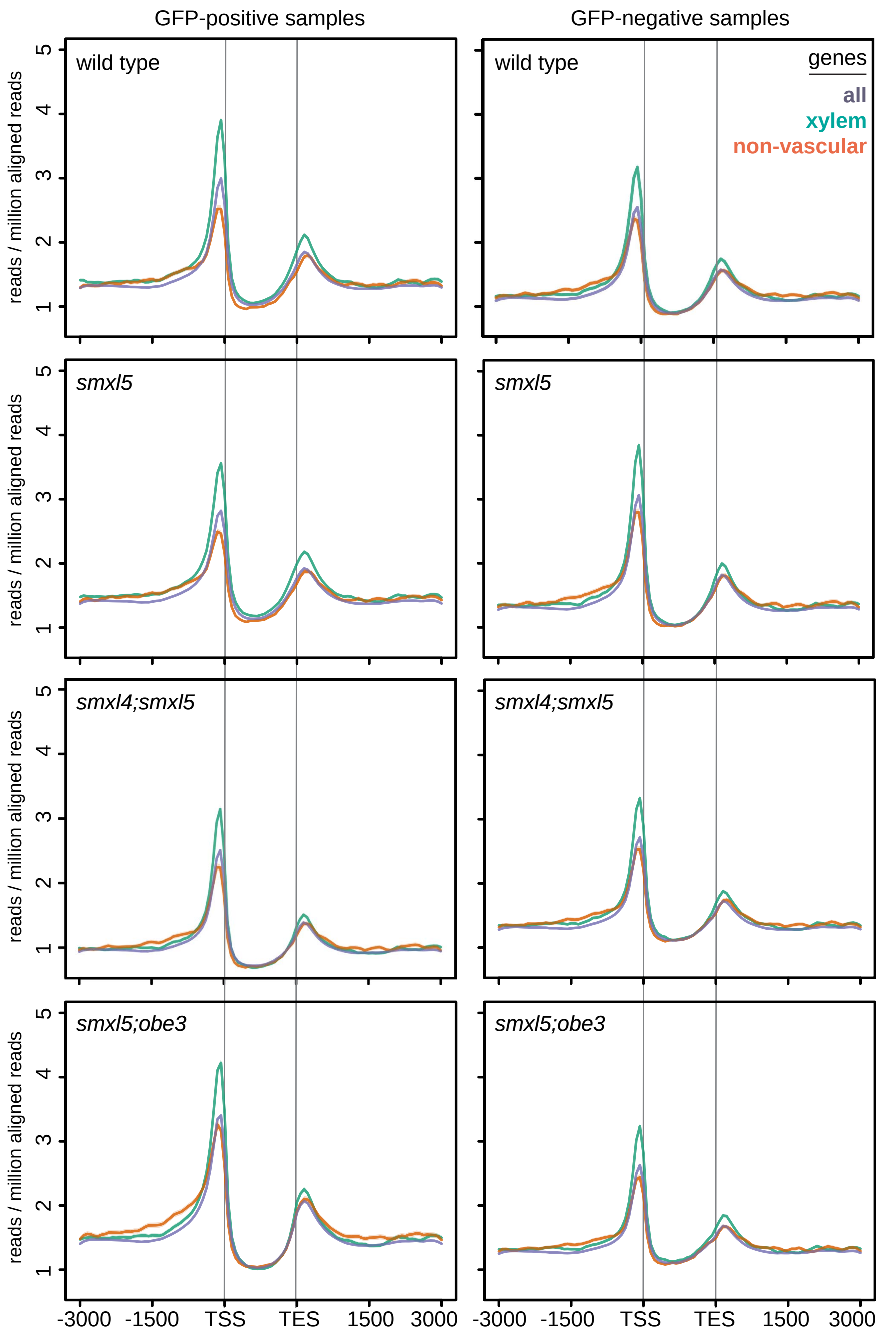

Supplementary Fig. 11: Chromatin profile of xylem-related and non-vascular genes
Chromatin conformation profiles for all genes found in the Arabidopsis genome or only for xylem-related and non-vascular genes according to Brady et al., 2007 in phloem and non-phloem-related cells comparing different genetic backgrounds. Read alignment was adjusted to transcriptional start sites (TSS) and transcriptional end sites (TES). X-axes show distance to TSSs and TESs in base-pairs.

Supplementary Table 1: Oligonucleotides used in this study

	Used for	Primer name	Primer sequence (5' → 3')
Genotyping	*smxl3-1*	*SALK_024706_LP*	CCCTACACAGCTCTTCACGAG
		SALK_024706_RP	TGCCTCTCTCACAAGAAAAGC
	smxl4-1	*SALK_037136-LP*	TTGAAGCCATGGAAGAATCTG
		SALK_037136-RP	ACAAAGAACAATGCGGTCAAG
	smxl5-1	*SALK_018522-LP*	TGTCTCATTGAAGCCAAAACC
		SALK_018522-RP	AATGGTGCAAGAATTCTGACG
	obe1-1	*SALK_075710_LP*	ATTCGACTCAAACGTTGAACG
		SALK_075710_RP	CTCGTCTGGACAAACTTCTGC
	obe2-1	*obe2-1_RP*	CTTCAAGATCAAGGTATTGACCTAAATTACC
		obe2-1_LP	CATTTGGTGAGGATGATTCGAACC
		obe2-1_Insertion	GATCAGATTGTCGTTTCCCGCC
	obe3-1	*SALK_042597_LP*	TTCCAACAACAAAGGCTTTTG
		SALK_042597_RP	TTTCCCACAAAACGAAACAAG
	obe4-1	*SALK_082338_LP*	TGCTTATTGACACCTGACTGC
		SALK_082338_RP	AAGAAAAGCGAGGAGGAAGTG
	SALK_insert	*SALK_LBa1*	TGGTTCACGTAGTGGGCCATCG
Molecular cloning			
	pEW31	*3xHA_for*	ACTAGGATCCTCCGCTTCCTCCTGCGTAGTCCGGGACATCATAC
		3xHA_rev	ACTAAAGCTTATGTATCCTTATGATGTACCTGATTATG
	pEW33	*3xHA_SMXL5_for*	ACTAGGATCCAGGAGGAAGTGGAGGAAGTTATCCTTATGATGTACCTG
		3xHA_SMXL5_rev	ACTACCCGGGTCATGCGTAGTCCGGGACATC
	miRNA PCRs for *pEW45/46*	*P-0950 Oligo A*	AACAGGTCTCAAACACTGCAGCCCCAAACACACGC
		P-0951 Oligo B	AACAGGTCTCTGCAGCCCCATGGCGATGCC
	pEW45	*I_miRNAsOBE3_3*	GATAATTTCTGGTATTGACTCAGTCTCTCTTTTGTATTCC
		II_miRNAaOBE3_3	GACTGAGTCAATACCAGAAATTATCAAAGAGAATCAATGA
		*III_miRNA*sOBE3_3*	GACTAAGTCAATACCTGAAATTTTCACAGGTCGTGATATG
		*IV_miRNA*aOBE3_3*	GAAAATTTCAGGTATTGACTTAGTCTACATATATATTCCT
	pEW46	*I_miRNAsOBE3_4*	GATAATTTCTGGTATTGACTCATTCTCTCTTTTGTATTCC
		II_miRNAaOBE3_4	GAATGAGTCAATACCAGAAATTATCAAAGAGAATCAATGA
		*III_miRNA*sOBE3_4*	GAATAAGTCAATACCTGAAATTTTCACAGGTCGTGATATG
		*IV_miRNA*aOBE3_4*	GAAAATTTCAGGTATTGACTTATTCTACATATATATTCCT
	pKG33	*CEB1for4*	ACTATCTAGAATGCGAACAGGTGGTTATACGATTC
		CEB1rev13	ACTAGGATCCTCGAACTTGGAAACTTGG
	pKG34	*YFP-CEB1*	ACTAGGATCCGGGAGGAAGTGGAGTGAGCAAGGGCGAGGAGC
		YFP/CFPrev7	ACTACCCGGGTCACTTGTACAGCTCGTCCATG

Supplementary Table 2: Resources used in this study

RESOURCE	SOURCE	IDENTIFIER
Antibodies		
Anti-HA-Peroxidase High Affinity (3F10), rat monoclonal	Roche; Basel, Switzerland	Cat# 11867423001
c-Myc Antibody sc-40 HRP (9E10), mouse monoclonal	Santa Cruz Biotechnology, Santa Cruz, USA	Cat# sc-40
Bacterial and Virus Strains		
Agrobacterium tumefaciens C58C1: RifR with pSoup plasmid (TetR)	ref[3], ref[4]	
Agrobacterium tumefaciens ASE: KanR, CamR with pSoup+ plasmid (TetR)	ref[5], ref[4]	
Agrobacterium tumefaciens C58C1: RifR with *35S:P19*	ref[6]	
Chemicals, Peptides, and Recombinant Proteins		
DirectRed 23	Sigma-Aldrich; St. Louis, USA	Cat#212490
ClearSee	ref[7]	
Propidium iodide	Sigma-Aldrich; St. Louis, USA	Cat#81845
Nicotiana infiltration induction buffer (10 mM MES pH 5.5, 10 mM MgSO4, 150 µM Acetosyringone)	This paper	N/A
cOmplete™ Protease Inhibitor Cocktail	Sigma-Aldrich; St. Louis, USA	Cat#11697498001
Protein extraction buffer (50 nM Na_3PO_4, 150 mM NaCl, 10 % Glycerol, 5 mM EDTA, 10 µM β-mercaptoethanol, 0.1 % Triton X-100, 2 mM $NaVO_4$, 2 mM NaF, cOmplete tablet (1/2 tablet per 20ml), 20 µM MG132, 1 mM PMSF)	This paper	N/A
Wash buffer I (50 nM Na_3PO_4, 150 mM NaCl, 10 % Glycerol, 5 mM EDTA, 0.1 % triton X-100, 2 mM $NaVO_4$, 2 mM NaF, cOmplete tablet (1/2 tablet per 20ml), 20 µM MG132, 1 mM PMSF)	This paper	N/A
Wash buffer II (50 nM Na_3PO_4, 150 mM NaCl, 10 % Glycerol, 5 mM EDTA)	This paper	N/A
Sample Buffer, Laemmli 2x Concentrate	Sigma-Aldrich; St. Louis, USA	Cat#S3401
SuperSignal™ West Femto Maximum Sensitivity Substrate	Thermo-Scientific; Waltham, USA	Cat#34094
PageRuler™ Plus Prestained Protein Ladder, 10 to 250 kDa	Thermo-Scientific; Waltham, USA	Cat#26619
Bio-Rad Protein Assay Dye Reagent Concentrate	Bio-Rad Laboratories; Hercules, USA	Cat#5000006EDU

Experimental Models: Organisms/Strains		
Arabidopsis: wild type Col-0	Gudrun Böhmdorfer	
Arabidopsis: *smxl5-1 (SALK_018522)*	ref[8]	N/A
Arabidopsis: *smxl4-1 (SALK_037136)*	ref[8]	N/A
Arabidopsis: *smxl3-1 (SALK_024706)*	ref[8]	N/A
Arabidopsis: *obe1-1 (SALK_075710)*	ref[9]	N/A
Arabidopsis: *obe2-1 (KG16805)*	ref[9]	N/A
Arabidopsis: *obe3-2/tta1-2 (SALK_042597)*	ref[10]	N/A
Arabidopsis: *obe4-1/tta2-1 (SALK_082338)*	ref[10]	N/A
Arabidopsis: *smxl4-1;smxl5-1 (SALK_037136, SALK_018522)*	ref[8]	N/A
Arabidopsis: *smxl4-1;obe1-1 (SALK_037136, SALK_075710)*	This paper	N/A
Arabidopsis: *smxl5-1;obe1-1 (SALK_018522, SALK_075710)*	This paper	N/A
Arabidopsis: *smxl4-1;obe2-1 (SALK_037136, KG16805)*	This paper	N/A
Arabidopsis: *smxl5-1;obe2-1 (SALK_018522, KG16805)*	This paper	N/A
Arabidopsis: *smxl4-1;obe3-2 (SALK_037136, SALK_042597)*	This paper	N/A
Arabidopsis: *smxl5-1;obe3-2 (SALK_018522, SALK_042597)*	This paper	N/A
Arabidopsis: *smxl3-1;obe3-2 (SALK_024706, SALK_042597)*	This paper	N/A
Arabidopsis: *smxl4-1;obe4-1 (SALK_037136, SALK_082338)*	This paper	N/A
Arabidopsis: *smxl5-1;obe4-1(SALK_018522, SALK_082338)*	This paper	N/A
Arabidopsis: brx-2 (pSKI15)	ref[11]	N/A
Arabidopsis: ops-2 (SALK_139316)	ref[12]	N/A
Arabidopsis: brx-2;smxl5-1 (pSKI15, SALK_018522)	This paper	N/A
Arabidopsis: ops-2;smxl5-1 (SALK_139316, SALK_018522)	This paper	N/A
Arabidopsis: *SMXL4:SMXL4-YFP* in wild type	ref[8]	N/A
Arabidopsis: *OBE3:OBE3-GFP/TTA1:TTA1-GFP* in wild type	ref[10]	N/A
Arabidopsis: *OBE4:OBE4-GFP/TTA2:TTA2-GFP* in wild type	ref[10]	N/A
Arabidopsis: SMXL5:OBE3-turquoise in *smxl5-1 obe3-2 (SALK_018522, SALK_042597)*	This paper	N/A
Arabidopsis: SMXL5:obe3-miRNA3 in *smxl5-1 (SALK_018522)*	This paper	N/A
Arabidopsis: SMXL5:obe3-miRNA4 in *smxl5-1 (SALK_018522)*	This paper	N/A
Arabidopsis: OPS:SMXL5-VENUS (pNT52) in *smxl4-1 smxl5-1 (SALK_037136, SALK_018522)*	This paper	N/A
OPS:ER-VENUS (pNT53) in wild type	This paper	N/A

BAM3:SMXL5-VENUS (pNT49) in *smxl4-1;smxl5-1 (SALK_037136, SALK_018522)*	This paper	N/A
BAM3:ER-VENUS (pNT50) in wild type	This paper	N/A
CVP2:SMXL5-VENUS (pNT16) in *smxl4-1;smxl5-1 (SALK_037136, SALK_018522)*	This paper	N/A
CVP2:ER-VENUS (pNT69) in wild type	This paper	N/A
APL:SMXL5-VENUS (pNT10) in *smxl4-1;smxl5-1 (SALK_037136, SALK_018522)*	This paper	N/A
APL:ER-VENUS (pNT68) in wild type	This paper	N/A
SMXL4:BRX-VENUS (pNT72) in *smxl4-1;smxl5-1 (SALK_037136, SALK_018522)*	This paper	N/A
OPS:OPS-GFP in wild type	ref[13]	N/A
OPS:OPS-mGFP6 in *smxl4-1;smxl5-1 (SALK_037136, SALK_018522)*	This paper	N/A
BRX:BRX-CITRINE in wild type	ref[13]	N/A
BRX:BRX- CITRINE in *smxl4-1;smxl5-1 (SALK_037136, SALK_018522)*	This paper	N/A
BAM3:BAM3- CITRINE in wild type	ref[13]	N/A
BAM3:BAM3- CITRINE in *smxl4-1;smxl5-1 (SALK_037136, SALK_018522)*	This paper	N/A
CVP2:NLS-VENUS in wild type	ref[13]	N/A
CVP2:NLS-VENUS in *smxl4-1;smxl5-1 (SALK_037136, SALK_018522)*	This paper	N/A
Nicotiana benthamiana	Karin Schumacher	N/A
*Saccharomyces cerevisiae: AH109 (*MATa, trp1-901, leu2-3, 112, ura3-52, his3-200, gal4Δ, gal80Δ, LYS2 : : GAL1UAS-GAL1TATA-HIS3, GAL2UAS-GAL2TATA-ADE2, URA3 : : MEL1UAS-MEL1 TATA-lacZ)	Armin Djamei Matchmaker^{TM} Two-Hybrid System 3 (Clontech, Palo Alto)	https://www.takarabio.com/assets/documents/User%20Manual/PT3247-1.pdf
Recombinant DNA		
35S:SMXL5-3xHA	This paper	pEW33
35S:6xc-Myc-OBE3	This paper	*pEW78*
35S*:OBE3-mGFP*	This paper	*pVL127*
35S:SMXL5-mCherry	This paper	*pVL122*
UBI10:mGFP-mCherry-NLS	This paper	*pCW194*
35S:mCherry-NLS	This paper	*pMG103*
SMXL5:OBE3-miRNA3	This paper	*pEW65*
SMXL5:OBE3-miRNA4	This paper	*pEW66*
SMXL4:SMXL4-YFP (+ 3' *SMXL4* terminator)	ref[8]	*pEW23*
SMXL5:OBE3-turquoise	This paper	*pEW72*
OPS:SMXL5-VENUS	This paper	*pNT52*
OPS:ER-VENUS	This paper	*pNT53*
BAM3:SMXL5-VENUS	This paper	*pNT49*
BAM3:ER-VENUS	This paper	*pNT50*
CVP2:SMXL5-VENUS	This paper	*pNT16*
CVP2:ER-VENUS	This paper	*pNT69*
APL:SMXL5-VENUS	This paper	*pNT10*
APL:ER-VENUS	This paper	*pNT68*
SMXL4:BRX-VENUS	This paper	*pNT72*

BRX:BRX-CITRINE	ref[13]	*N/A*
Software		
WMD3 – Web MicroRNA Designer Version 3	Max Planck Institute for Developmental Biology, Tübingen.	http://www.weigelworld.org
CLC Main Workbench 7.6.1	CLC Bio Qiagen, Aarhus, Denmark	
ImageJ 1.51h	National Institute of Health, Bethesda, USA	
Microsoft Office 2016	Microsoft, Redmond, USA	
Adobe Creative Suite CS6	Adobe, San Jose, USA	
SPSS V. 25	IBM, Armonk, USA	
GraphPad Prism version 6.01	GraphPad Software, La Jolla, USA	
Other		
Anti-HA MicroBeads	Miltenyi Biotec, Bergisch Gladbach, Germany	Cat#130-094-255
µ Columns	Miltenyi Biotec, Bergisch Gladbach, Germany	Cat#130-042-701
Advanced Fluorescence and ECL Imager	Intas Science Imaging Instruments, Göttingen, Germany	N/A
Confocal microscope TCS SP5	Leica Microsystems; Mannheim, Germany	N/A
Confocal microscope TCS SP8	Leica Microsystems; Mannheim, Germany	N/A

Supplementary References

1 Kosugi, S., Hasebe, M., Tomita, M. & Yanagawa, H. Systematic identification of cell cycle-dependent yeast nucleocytoplasmic shuttling proteins by prediction of composite motifs. *Proceedings of the National Academy of Sciences of the United States of America* **106**, 10171-10176, doi:10.1073/pnas.0900604106 (2009).

2 Brady, S. M. *et al.* A high-resolution root spatiotemporal map reveals dominant expression patterns. *Science* **318**, 801-806 (2007).

3 Ashby, A. M., Watson, M. D., Loake, G. J. & Shaw, C. H. Ti plasmid-specified chemotaxis of Agrobacterium tumefaciens C58C1 toward vir-inducing phenolic compounds and soluble factors from monocotyledonous and dicotyledonous plants. *Journal of bacteriology* **170**, 4181-4187 (1988).

4 Hellens, R. P., Edwards, E. A., Leyland, N. R., Bean, S. & Mullineaux, P. M. pGreen: a versatile and flexible binary Ti vector for Agrobacterium-mediated plant transformation. *Plant molecular biology* **42**, 819-832 (2000).

5 Merritt, C. D., Raina, S., Fedoroff, N. & Curtis, W. R. Direct Agrobacterium tumefaciens-mediated transformation of Hyoscyamus muticus hairy roots using green fluorescent protein. *Biotechnology progress* **15**, 278-282 (1999).

6 Voinnet, O., Rivas, S., Mestre, P. & Baulcombe, D. An enhanced transient expression system in plants based on suppression of gene silencing by the p19 protein of tomato bushy stunt virus. *The Plant journal : for cell and molecular biology* **33**, 949-956 (2003).

7 Kurihara, D., Mizuta, Y., Sato, Y. & Higashiyama, T. ClearSee: a rapid optical clearing reagent for whole-plant fluorescence imaging. *Development (Cambridge, England)* **142**, 4168-4179 (2015).

8 Wallner, E. S. *et al.* Strigolactone- and Karrikin-Independent SMXL Proteins Are Central Regulators of Phloem Formation. *Current biology : CB* **27**, 1241-1247 (2017).

9 Saiga, S. *et al.* The Arabidopsis OBERON1 and OBERON2 genes encode plant homeodomain finger proteins and are required for apical meristem maintenance. *Development (Cambridge, England)* **135**, 1751-1759 (2008).

10 Saiga, S. *et al.* Control of embryonic meristem initiation in Arabidopsis by PHD-finger protein complexes. *Development (Cambridge, England)* **139**, 1391-1398 (2012).

11 Rodrigues, A. *et al.* The short-rooted phenotype of the brevis radix mutant partly reflects root abscisic acid hypersensitivity. *Plant physiology* **149**, 1917-1928 (2009).

12 Truernit, E., Bauby, H., Belcram, K., Barthelemy, J. & Palauqui, J. C. OCTOPUS, a polarly localised membrane-associated protein, regulates phloem differentiation entry in Arabidopsis thaliana. *Development (Cambridge, England)* **139**, 1306-1315 (2012).

13 Rodriguez-Villalon, A. *et al.* Molecular genetic framework for protophloem formation. *Proceedings of the National Academy of Sciences of the United States of America* **111**, 11551-11556 (2014).
