# Supplementary material for: OBERON3 and SUPPRESSOR OF MAX2 1-LIKE proteins form a regulatory module driving phloem development
Source: Nat Commun. 2023 Apr 14;14:2128. doi: 10.1038/s41467-023-37790-5 (PMC10104830; doi:10.1038/s41467-023-37790-5)
Supplement: Supplementary file 3 — Description of Additional Supplementary Files [file 41467_2023_37790_MOESM3_ESM.pdf]

Description of Additional Supplementary Files:

Supplementary Data 1: Sequence of clones from OBE genes isolated during the yeast-two-hybrid screen using the SMXL5 protein as a bait.

Supplementary Data 2: OCRs and associated genes specific for GFP-positive or GFP-negative fractions from wild type, smxl5, smxl4;smxl5 or smxl5;obe3 backgrounds, respectively.

Supplementary Data 3: Genes with OCRs specific for GFP-positive or GFP-negative fractions for wild type, smxl5, smxl4;smxl5 and smxl5;obe3 backgrounds.

Supplementary Data 4: OCRs and associated genes specific for wild type, smxl5, smxl4;smxl5 and smxl5;obe3 plants comparing GFP-positive fractions from the respective backgrounds.

Supplementary Data 5: OCRs and associated genes specific for wild type, smxl5, smxl4;smxl5 and smxl5;obe3 plants comparing GFP-negative fractions from the respective backgrounds.

Supplementary Data 6: Xylem, phloem and non-phloem genes according to Brady et al., 2007.
