# Supplementary material for: OBERON3 and SUPPRESSOR OF MAX2 1-LIKE proteins form a regulatory module driving phloem development
Source: Nat Commun. 2023 Apr 14;14:2128. doi: 10.1038/s41467-023-37790-5 (PMC10104830; doi:10.1038/s41467-023-37790-5)
Supplement: Supplementary file 10 — Reporting Summary [file 41467_2023_37790_MOESM10_ESM.pdf]

nature portfolio

Corresponding author(s): Thomas Greb

Last updated by author(s): Mar 14, 2023

Reporting Summary

Nature Portfolio wishes to improve the reproducibility of the work that we publish. This form provides structure for consistency and transparency in reporting. For further information on Nature Portfolio policies, see our Editorial Policies and the Editorial Policy Checklist.

Statistics

For all statistical analyses, confirm that the following items are present in the figure legend, table legend, main text, or Methods section.

n/a	Confirmed	
☐	☒	The exact sample size (*n*) for each experimental group/condition, given as a discrete number and unit of measurement
☐	☒	A statement on whether measurements were taken from distinct samples or whether the same sample was measured repeatedly
☐	☒	The statistical test(s) used AND whether they are one- or two-sided<br>*Only common tests should be described solely by name; describe more complex techniques in the Methods section.*
☐	☒	A description of all covariates tested
☐	☒	A description of any assumptions or corrections, such as tests of normality and adjustment for multiple comparisons
☐	☒	A full description of the statistical parameters including central tendency (e.g. means) or other basic estimates (e.g. regression coefficient) AND variation (e.g. standard deviation) or associated estimates of uncertainty (e.g. confidence intervals)
☐	☒	For null hypothesis testing, the test statistic (e.g. *F*, *t*, *r*) with confidence intervals, effect sizes, degrees of freedom and *P* value noted<br>*Give P values as exact values whenever suitable.*
☒	☐	For Bayesian analysis, information on the choice of priors and Markov chain Monte Carlo settings
☒	☐	For hierarchical and complex designs, identification of the appropriate level for tests and full reporting of outcomes
☒	☐	Estimates of effect sizes (e.g. Cohen's *d*, Pearson's *r*), indicating how they were calculated

Our web collection on statistics for biologists contains articles on many of the points above.

Software and code

Policy information about availability of computer code

Data collection	Leica, image analysis software, ImageJ
Data analysis	bowtie2 (v2.2.6), picard MarkDuplicates (v2.25.5), bamCoverage in deepTools (v3.5.1), igv, homer (v4.10.3), bedtools (v2.27.1), ngs.plot (v2.47), IBM SPSS Statistics, GraphPad Prism version 6.01

For manuscripts utilizing custom algorithms or software that are central to the research but not yet described in published literature, software must be made available to editors and reviewers. We strongly encourage code deposition in a community repository (e.g. GitHub). See the Nature Portfolio guidelines for submitting code & software for further information.

Data

Policy information about availability of data

All manuscripts must include a data availability statement. This statement should provide the following information, where applicable:

- Accession codes, unique identifiers, or web links for publicly available datasets
- A description of any restrictions on data availability
- For clinical datasets or third party data, please ensure that the statement adheres to our policy

Raw ATAC-seq data were deposited to the NCBI GEO archive (Barrett et al. 2013) under the accession GSE184344 (https://www.ncbi.nlm.nih.gov/geo/query/acc.cgi?acc=GSE184344). Accession codes and unique identifiers of used material are mentioned in the Resource Table and the Material and Method section. All used material is available upon request to the corresponding author.

Human research participants

Policy information about studies involving human research participants and Sex and Gender in Research.

Reporting on sex and gender	n/a
Population characteristics	n/a
Recruitment	n/a
Ethics oversight	n/a

Note that full information on the approval of the study protocol must also be provided in the manuscript.

Field-specific reporting

Please select the one below that is the best fit for your research. If you are not sure, read the appropriate sections before making your selection.

☒ Life sciences ☐ Behavioural & social sciences ☐ Ecological, evolutionary & environmental sciences

For a reference copy of the document with all sections, see nature.com/documents/nr-reporting-summary-flat.pdf

Life sciences study design

All studies must disclose on these points even when the disclosure is negative.

Sample size	Sample sizes are indicated in the respective figure legends and have been maximized according to practical considerations. Sample size is >10 and usually higher.
Data exclusions	no data were excluded
Replication	All displayed data represent at least three independent, technical repetitions, unlike otherwise indicated.
Randomization	For practical reasons (enhanced physical handling of plants), randomization was not possible. Covariates were controlled by applying the exact same conditions (growth substrate, temperature, light) to all individuals.
Blinding	Blinding was not possible as phenotypic differences between lines allowed immediate identification.

Reporting for specific materials, systems and methods

We require information from authors about some types of materials, experimental systems and methods used in many studies. Here, indicate whether each material, system or method listed is relevant to your study. If you are not sure if a list item applies to your research, read the appropriate section before selecting a response.

Materials & experimental systems

n/a	Involved in the study	
☐	☒	Antibodies
☒	☐	Eukaryotic cell lines
☒	☐	Palaeontology and archaeology
☐	☒	Animals and other organisms
☒	☐	Clinical data
☒	☐	Dual use research of concern

Methods

n/a	Involved in the study	
☒	☐	ChIP-seq
☐	☒	Flow cytometry
☒	☐	MRI-based neuroimaging

Antibodies

Antibodies used	Anti-HA-Peroxidase High Affinity (3F10), rat monoclonal; c-Myc Antibody sc-40 HRP (9E10), mouse monoclonal
Validation	negative controls not containing the respective proteins did not show the repsective signals in Western analyses

Animals and other research organisms

Policy information about studies involving animals; ARRIVE guidelines recommended for reporting animal research, and Sex and Gender in Research

Laboratory animals	n/a
Wild animals	n/a
Reporting on sex	n/a
Field-collected samples	n/a
Ethics oversight	n/a

Note that full information on the approval of the study protocol must also be provided in the manuscript.

Flow Cytometry

Plots

Confirm that:

☒ The axis labels state the marker and fluorochrome used (e.g. CD4-FITC).

☒ The axis scales are clearly visible. Include numbers along axes only for bottom left plot of group (a 'group' is an analysis of identical markers).

☒ All plots are contour plots with outliers or pseudocolor plots.

☒ A numerical value for number of cells or percentage (with statistics) is provided.

Methodology

Sample preparation	Nucleus extraction for FANS/ATAC-seq was based on (Thibivilliers et al. 2020) and carried out on ice. Approximately 1 gr of three week-old Arabidopsis seedlings grown in ½ MS LD conditions were collected in a 60 mm petri dish and thoroughly chopped using a razor blade for five minutes in nucleus isolation buffer (1x NIB, CelLytic™-PN Isolation/Extraction buffer, Sigma-Aldrich, cat.no. CELLYTPN1) supplemented with 10 µg/ml Hoechst 33342 (Sigma-Aldrich, B2261) as the final concentration. After incubation for 15 min at 4°C in darkness, the nucleus suspension was applied to 50 µm nylon strainers mounted into a 30 µm nylon strainers at the top (Sysmex, CellTrics), and filtered further by a FACS tube with 35 µm strainer cap (Corning, #352235). 15,000 nuclei for each sample were then sorted according to their GFP signal levels into a 300 µL collection buffer (15 mM TRIS-HCl ph 7.5, 20 mM NaCl, 80 mM KCl, 0.1 % Triton) by a BD FACSAriaTM IIIu cell sorter using a 100 µm sort nozzle and a 30 kHz drop drive frequency.
Instrument	BD FACSAriaTM IIIu cell sorter
Software	BD FACSDiva™
Cell population abundance	At least 10,000 nuclei were sampled in each case. The gate for GFP+ nuclei was set using wild type as a reference.
Gating strategy	The gate for GFP+ nuclei was set using wild type as a reference.

☒ Tick this box to confirm that a figure exemplifying the gating strategy is provided in the Supplementary Information.
